# Supplementary material for: Colostrum Quality Assessment in Dairy Goats: Use of an On-Farm Optical Refractometer
Source: Biology (Basel). 2023 Apr 20;12(4):626. doi: 10.3390/biology12040626 (PMC10135588; doi:10.3390/biology12040626)
Supplement: Supplementary file 1 [file biology-12-00626-s001.zip › biology-2261082-supplementary.pdf]

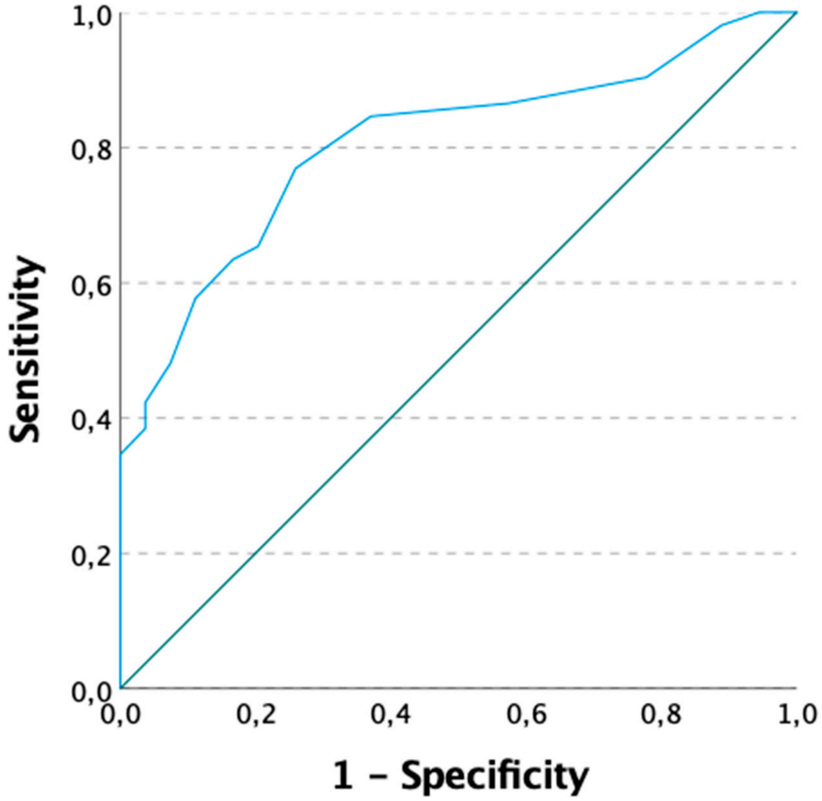

Figure S1. ROC curve for the detection of good-quality colostrum (based on cut-off threshold of 20 mg/ml as measured by ELISA) using an optical Brix refractometer to estimate the quality of fresh goat colostrum samples measured by ELISA.

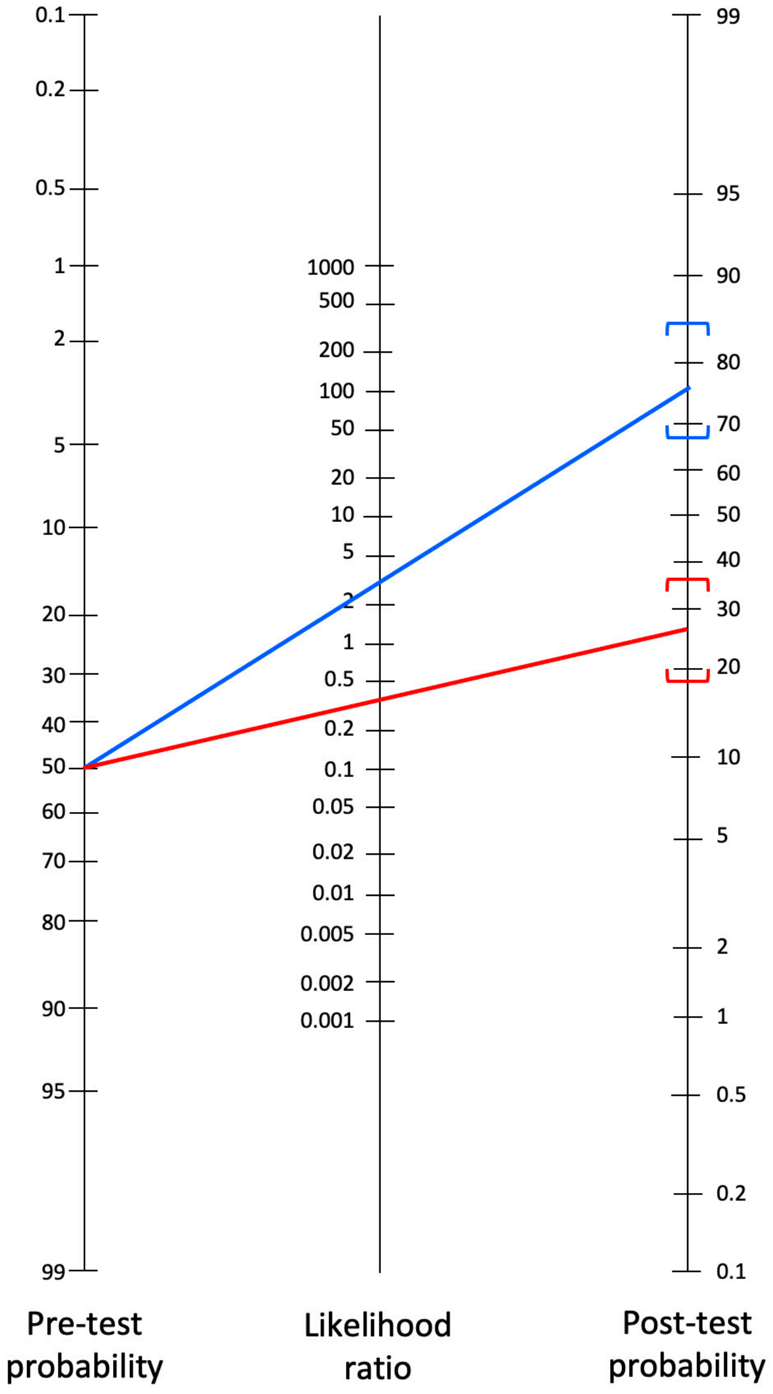

Figure S2. Fagan's nomogram to determine post-test probability of fresh colostrum Brix refractometer values detecting low IgG values (<20 mg/ml).

Table S1. Sensitivity, specificity and Youden index of Brix refractometer scores to predict colostrum IgG values higher than 20 mg/ml.

Cut-off value of Brix value	Sensitivity (%)	Specificity (%)	Youden index
11	100	0	0
12	100	5.6	0.056
13	98.1	11.1	0.092
14	90.4	22.2	0.126
15	86.5	42.6	0.291
16	84.6	63.0	0.476
17	80.8	68.5	0.493
18	76.9	74.1	**0.510**
19	65.4	79.6	0.450
20	63.5	83.3	0.468
21	57.7	88.9	0.466
22	48.1	92.6	0.407
23	42.3	96.3	0.386
24	38.5	96.3	0.348
25	34.6	100	0.346
26	26.9	100	0.269
27	19.2	100	0.192
28	15.4	100	0.154
29	13.5	100	0.135
30	11.5	100	0.115
31	9.6	100	0.096
32	7.7	100	0.077
33	5.8	100	0.058
34	3.8	100	0.038
35	1.9	100	0.019
35	0	100	0
